# Enhancing Maternal and Perinatal Health in Under-Served Remote Areas in Sub-Saharan Africa: A Tanzanian Model

**DOI:** 10.1371/journal.pone.0151419

**Published:** 2016-03-17

**Authors:** Angelo S. Nyamtema, Nguke Mwakatundu, Sunday Dominico, Hamed Mohamed, Senga Pemba, Richard Rumanyika, Clementina Kairuki, Irene Kassiga, Allan Shayo, Omary Issa, Calist Nzabuhakwa, Chagi Lyimo, Jos van Roosmalen

**Affiliations:** 1 World Lung Foundation—Maternal Health Project, Dar es Salaam, Tanzania; 2 Tanzanian Training Centre for International Health, Ifakara, Tanzania; 3 Saint Francis University College for Health and Allied Sciences, Ifakara, Tanzania; 4 Department of Obstetrics and Gynaecology, Catholic University of Health and Allied Sciences, Mwanza, Tanzania; 5 Hubert Kairuki Memorial University, Dar es Salaam, Tanzania; 6 Department of Obstetrics and Gynaecology, Saint Francis Referral Hospital, Ifakara, Tanzania; 7 Department of Obstetrics, Leiden University Medical Centre, Leiden, The Netherlands; 8 Athena Institute, VU University Amsterdam, Amsterdam, The Netherlands; University British Columbia, CANADA

## Abstract

**Background:**

In Tanzania, maternal mortality ratio (MMR), unmet need for emergency obstetric care and health inequities across the country are in a critical state, particularly in rural areas. This study was established to determine the feasibility and impact of decentralizing comprehensive emergency obstetric and neonatal care (CEmONC) services in underserved rural areas using associate clinicians.

**Methods:**

Ten health centres (HCs) were upgraded by constructing and equipping maternity blocks, operating rooms, laboratories, staff houses and installing solar panels, standby generators and water supply systems. Twenty-three assistant medical officers (advanced level associate clinicians), and forty-four nurse-midwives and clinical officers (associate clinicians) were trained in CEmONC and anaesthesia respectively. CEmONC services were launched between 2009 and 2012. Monthly supportive supervision and clinical audits of adverse pregnancy outcomes were introduced in 2011 in these HCs and their respective district hospitals.

**Findings:**

After launching CEmONC services from 2009 to 2014 institutional deliveries increased in all upgraded rural HCs. Mean numbers of monthly deliveries increased by 151% and obstetric referrals decreased from 9% to 3% (p = 0.03) in HCs. A total of 43,846 deliveries and 2,890 caesarean sections (CS) were performed in these HCs making the mean proportion of all births in EmONC facilities of 128% and mean population-based CS rate of 9%. There were 190 maternal deaths and 1,198 intrapartum and very early neonatal deaths (IVEND) in all health facilities. Generally, health centres had statistically significantly lower maternal mortality ratios and IVEND rates than district hospitals (p < 0.00 and < 0.02 respectively). Of all deaths (maternal and IVEND) 84% to 96% were considered avoidable.

**Conclusions:**

These findings strongly indicate that remotely located health centres in resource limited settings hold a great potential to increase accessibility to CEmONC services and to improve maternal and perinatal health.

## Introduction

Achieving universal access to reproductive health services and reducing maternal and perinatal mortality have remained the greatest challenges in sub-Saharan Africa, a region with the highest maternal mortality ratio (MMR) of 510/ 10^5^ live births [1]. Tanzania has the sixth highest number of maternal deaths in the world with 7,900 deaths annually and is one of the ten countries contributing to 58% of the global burden of maternal deaths [1]. Currently, Tanzania’s MMR is 410/ 10^5^ live births (with uncertainty range 250–660) while the neonatal mortality rate is 21/10^3^ live births. Only 50% of all pregnant women deliver in health facilities with a met need for emergency obstetric care lying low at 15–43% and caesarean section rate (CSR) at 4.5% [1,2,3,4,5].

Further, Tanzania has one of the lowest densities of skilled health providers and the weakest rural infrastructure in the world, elements which are critical to meeting the health-related needs of the population [6,7]. Specifically, the density of physicians is as low as 3 per 100,000 population and only 42% of births in rural areas are assisted by skilled personnel compared to 83% in urban areas, indicating serious health inequities across the country [4,8]. Only a small proportion of facilities in Tanzania (4 percent) provide caesarean delivery services. As expected, caesarean delivery services are available mainly in hospitals (79%) and in some health centres (13%). In general, two-thirds of all hospitals (67%) and less than half (41%) of all health centres have regular, uninterrupted electricity (i.e., the facility is connected to a central power grid, or has solar power or both, and power is routinely available during regular service hours), or have a functioning generator with fuel. Similarly, only one third (31%) of all rural health facilities have an improved water source in the facility (i.e., water is piped into the facility or onto facility grounds, or else water is from a public tap or standpipe, a tube well or borehole, a protected dug well, or protected spring or rain water), and the outlet from this source is within 500 metres of the facility [9].

Although there is worldwide consensus that quality comprehensive emergency obstetric and neonatal care (CEmONC) is key for ensuring maternal and perinatal survival and wellbeing, many women living in remote, rural parts of Tanzania are unable to access good-quality EmONC [1,10,11]. The obstacles to such services include physical barriers of poor roads and long distances, financial barriers as well as poor quality of services [12,13,14]. In addressing these obstacles, one of the national strategies to accelerate the reduction of maternal and perinatal mortality focuses on upgrading 50% of the 684 existing health centres (HCs) in the country to provide CEmONC using nurse-midwives and clinical officers (associate clinicians) and assistant medical officers (AMOs) who are advanced level associate clinicians [15,16]. The World Health Organization (WHO) defines associate clinician as a professional clinician with basic competencies to diagnose and manage common medical, maternal, child health and surgical conditions [16]. In Tanzania, nurse-midwives and clinical officers are generally trained for 3 to 4 years post-secondary education in established higher education institutions. Advanced level associate clinician is defined as a professional clinician with advanced competencies to diagnose and manage the most common medical, maternal, child health and surgical conditions, including obstetric and gynaecological surgery (e.g. caesarean sections). In Tanzania, AMOs are clinical officers with an additional two years of formal training in clinical medicine which includes 3 months in surgery and 3 months in obstetrics and gynaecology.

With a goal to support national efforts to address this crisis, the World Lung Foundation (WLF) Maternal Health Project, co-funded by Bloomberg Philanthropies and Foundation H&B Agerup, designed and implemented a project that upgraded nine extremely remote HCs and one urban HC, while at the same time supporting respective district hospitals, to improve CEmONC services. This innovation aimed at formulating a model solution for maternal health care in rural settings. To study the implementation of this project, an operations research study was conducted. This article describes the results from this study including five years of project experience related to its implementation and the lessons learned.

## Materials and Methods

### Project areas

Administratively, Tanzania is subdivided into regions, districts, wards and villages. A ‘ward’ is composed of three to five villages and is supposed to have a health centre which serves a population of about 50,000 [17]. The project purposively selected and upgraded ten HCs in three regions (Kigoma, Morogoro and Pwani), of which nine were located in the hardest to reach rural areas in seven districts in Tanzania. The hospitals related to the HCs were also supported. Ujiji HC, the only urban health centre, was upgraded to decongest the high number of deliveries at Maweni regional referral hospital in Kigoma. The total population served by these health facilities as per 2012 population census was 1,771,350 [18]. Three of the selected HCs were located further than 150 km from their nearby hospitals with CEmONC services. As a result of long distances and/or poor roads, expectant mothers with obstetric complications spent up to 18 hours reaching hospitals with CEmONC services. During rainy seasons district hospitals were even more difficult to reach.

### Study Design

The World Health Organization defines operations research in reproductive health as research aimed to provide evidence-based scientific data and appropriate technologies to improve service delivery and utilization, and recommend relevant policies [19,20]. For this study, an operations research protocol based on the project design was created to produce results that could contribute to the development of solutions to current operational problems in maternal health, and to improve service delivery and increase utilization of CEmONC services in underserved rural areas in Tanzania. The project was designed to maximize the use of existing materials and human resources for maternal and perinatal health in rural areas. In order to optimize its impact, several interventions were implemented.

First, upgrading health facilities’ infrastructure, equipment and consumables: from 2008–2010 the project constructed new maternity blocks, operation theatres, laboratories and either constructed and/ or renovated staff houses. All facilities received basic essential equipment and the project complemented government supplies with pharmaceutical supplies and drugs in order to avoid stock outs. Solar panels and standby generators were also installed in seven HCs; three HCs already had connections to the national electricity grid. Water supply systems were also strengthened. Project support was guided by the results of a facility assessment which included a checklist of basic requirements for CEmONC services that was developed by the project team. The assessment looked at physical infrastructure, the availability of skilled health professionals, equipment and essential pharmaceutical supplies. CEmONC services were introduced in a facility only when the project team was satisfied that all basic requirements were in place.

Second, human resources capacity building: knowing that the provision of CEmONC services requires a team approach, assistant medical officers were trained in CEmONC and nurse/midwives and clinical officers were trained to provide anaesthesia. A detailed description of the training in CEmNOC and anaesthesia has been reported elsewhere [21]. Post course training sessions were carried out so as to strengthen knowledge and skills of care providers.

Third, supportive supervision, mentoring and clinical audits: following the introduction of CEmONC services, monthly supportive supervision and onsite mentoring were carried out by either an obstetrician or a more experienced AMO for 2–3 days in each HC. During these visits, training for *c*ontinuing professional development and clinical audits were carried out. Audit topics were: maternal deaths, cases of severe maternal morbidity (including severe pre–eclampsia and eclampsia, uterine rupture, antepartum and postpartum haemorrhage and puerperal sepsis], and intrapartum and very early neonatal deaths (IVEND). Audited cases was assessed based on national guidelines for management of emergency obstetric conditions. Impact of the project was based on the comparison of pre and post intervention results including: the monthly institutional deliveries, caesarean section, referrals, and numbers of maternal, intrapartum and very early neonatal deaths.

To supplement the health centre upgrades, the project also strengthened related district hospitals’ ability to provide CEmONC. The support included human resource capacity building, provision of essential equipment and pharmaceutical supplies and infrastructure improvement where necessary. Like in health centres, human resource capacity building included training of care providers in anaesthesia, CEmONC, supportive supervision and clinical audits. Prior to the project interventions, the five supported hospitals were already providing CEmONC services but issues related to the quality of care being delivered were identified as needing improvement. The outcomes of interest were changes in utilization of CEmONC services, changes in obstetric referral and changes in maternal and perinatal mortality.

### Development and Validation of Data Collection Forms

Data collection and audit record forms were developed and reviewed by a panel of five experienced obstetricians for relevance and clarity. Items regarded as relevant for inclusion were retained and inappropriate items were either removed or modified based on discussion. Data collection forms which were used to assess performance of health facilities had three main sections. Section one contained background information on the facility by month and year; section two collected routine indicators; and, section three included descriptions of problems encountered during implementation of the intervention in that particular month. Routine indicators included the number of: deliveries, vacuum deliveries, caesarean sections, referred women out of the facility (including reason for referral), maternal deaths, intrapartum deaths, stillbirths and very early neonatal deaths (within 24 hours of birth).

The audit form was piloted in early 2011 and was revised based on feedback from the review team. Patient information collected for audit included age, gravidity, parity, description of obstetric complications, antenatal care history, previous obstetric history, and intrapartum and postpartum care (where necessary). This information was collected strictly to enhance audit discussions, establish the cause of death, identify areas of substandard care and plan for future improvement.

### Data Collection

To determine the effectiveness of the interventions and performance of supported health facilities data were collected based on the year of introduction of CEmONC services in the respective health centre (i.e. one year before and after introduction of services). Sources of data included delivery records books, case files, partographs and operation records books. In case of missing information, staff who attended the patients were asked for clarification. A process of audit involved, focal person summarizing the cases for audit, the audit team critically reviewing the management of the cases to establish the cause of death or severe morbidity and underlying substandard care, and devising strategic action plans.

Areas of substandard care were classified using the Three Delays Model (i.e. Delay 1: delay in decision to seek care; Delay 2: delay in reaching care; and, Delay 3: delay in receiving adequate and appropriate health care) [22]. Strategic interventions aimed at improving care included focused training and replenishment of essential supplies, drugs and equipment.

### Ethics and Permission

Permission and approval to conduct research was obtained from the Ministry of Health and Social Welfare, the Prime Minister’s Office, and Local Government officials. There was no need for patient’s consent because this study was not designed to collect individual patient’s records and no author had direct interaction with patients at any point in time. During clinical audits, patients’ names were anonymised by health providers at the facility. Focal persons at the facilities completed case histories in the audit record forms. Since the research conducted related directly to rectifying urgent service delivery problems and was based on routine health care operations, this study did not require Institutional Review Board approval. However, all national and international ethical considerations were observed during implementation of the research.

### Data Analysis

Data collected were cleaned and consistency checks were done using Microsoft Excel. Data analysis was done using Stata (version 12). Impacts of this model were determined using trends of mean monthly deliveries and rates of caesarean sections, referrals, maternal, intrapartum and very early neonatal deaths. ANOVA (F-Test) analysis was performed to compare rates of these parameters by period i.e. the statistical differences of the rates before and after CEmONC intervention. These parameters were statistically compared using ANOVA (F-Test) with p-values at 0.05. When the null hypothesis in a one-way ANOVA was rejected, Scheffe’s method of pair wise multiple comparison was performed in order to identify which periods were the rates different from each other.

## Results

### Training of CEmONC Teams

**Fig 1:**
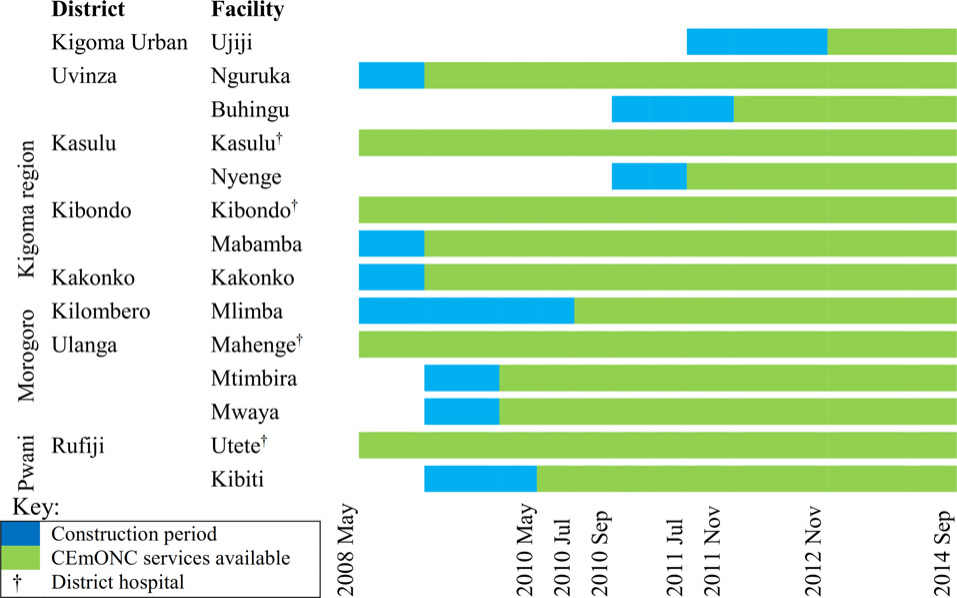
Timeline for facility construction and introduction of CEmONC services in WLF Supported Health Facilities.

From 2009 to February 2012 a total of 67 associate clinicians from the WLF supported health facilities were trained in CEmOC and anaesthesia in 7 batches at Maweni Regional Hospital in Kigoma and Tanzanian Training Centre for International Health/St Francis Regional Referral Hospital in Ifakara, Morogoro, Tanzania. Thirty-four percent of trainees were assistant medical officers from health centres who were trained in CEmOC, 52% were nurse-midwives trained in anaesthesia, and 13% were clinical officers also trained in anaesthesia. Of the trainees in anaesthesia (nurse-midwives and clinical officers), eight (18%) came from the supported district hospitals (i.e. Utete and Mahenge) and Maweni regional hospital in Kigoma. Training of staff was carried out before and after introduction of CEmOC services in health centres to ensure health providers’ confidence and competence. CEmONC services were introduced in the upgraded HCs between 2009 and 2012 (Fig 1).

### Process Indicators

**Fig 2:**
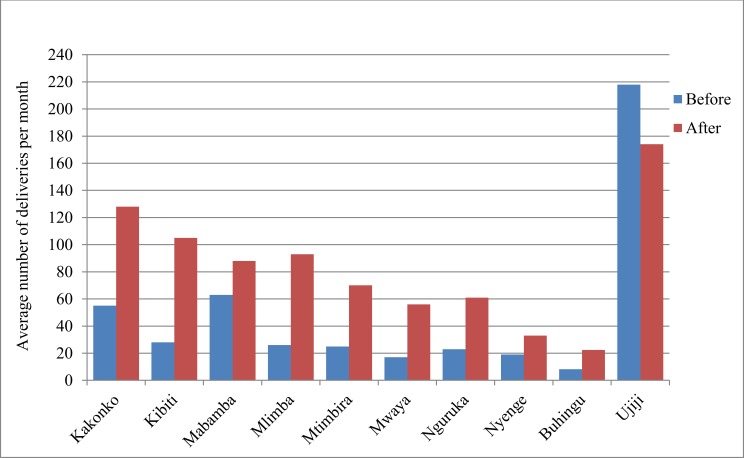
Mean monthly deliveries before and after introduction of CEmONC services in WLF supported health centres.

During the project period (2009 to 2014) a total of 91,196 deliveries took place in the supported health facilities. All upgraded rural health centres experienced significant increases in the number of women coming for delivery care after they began providing CEmONC services (Fig 2). On average, within the first year of delivering CEmONC services, the number of monthly deliveries increased by 151% ranging from 74% at Nyenge to as high as 320% at Kibiti. Of these, Mwaya, Mlimba and Kibiti experienced the highest increase of monthly deliveries ranging from 226% to 320%.

The mean population-based proportion of all births which took place in rural EmONC facilities was 128% (ranging from 42% at Buhingu to as high as 230% at Mwaya) (Table 1). It was learned through anecdotal reports that many expectant mothers from other ‘wards’ (outside the catchment area) also began using supported facilities. During the project period (2009 to 2014) a total of 2,890 caesarean sections out of 43,846 deliveries (6.6%) were performed in these health centres. The average proportion of cesarean sections as a proportion of all births in the catchment area served by upgraded rural HCs was 9%, reaching the WHO recommended range of 5% to 15% [10].

**Table 1:** Population based C-section rate after introducing CEmONC services in health facilities under WLF support.

Health Facility	Ward(s)-population served by the facility*	Expected births per year in 2012** in catchment area	Births in EmOC health facilities in 2012	Proportion of all births in EmONC facilities	Annual average No. of C-Sections (2012–2013)	Population based C-section rate (using # of expected births per year)
Kakonko	21,195	827	1,620	196%	48	6%
Mabamba	17,580	686	1,034	151%	25	4%
Nguruka	27,179	1,060	860	81%	43	4%
Nyenge	16,345	637	400	63%	9	1%
Mlimba	38,108	1,486	1,604	108%	183	12%
Mtimbira	16,000	624	1,038	166%	91	15%
Mwaya	8,763	342	786	230%	88	26%
Kibiti	15,156	591	1,198	203%	85	14%
Buhingu	16,973	662	277	42%	29	4%
Kasulu†	67,704	2,640	NA	NA	544	21%
Kibondo†	39,300	1,533	NA	NA	290	19%
Utete†	18,083	705	NA	NA	227	32%
Mahenge†	38,243	1,491	NA	NA	189	13%
Maweni†† & Ujiji HC	215,458	8,403	NA	NA	505	6%

Note

* Based on 2012 population and housing census by NBS

** expected number of births during 12 months, given the crude birth rate (39 live births per 1000 population) documented by NBS, 2010

† district hospitals

†† regional hospital. Proportions of all births in EmOC facilities were not estimated for the district hospitals’ catchment areas as there were other facilities which provided basic EmOC services, hence not applicable (NA).

**Fig 3:**
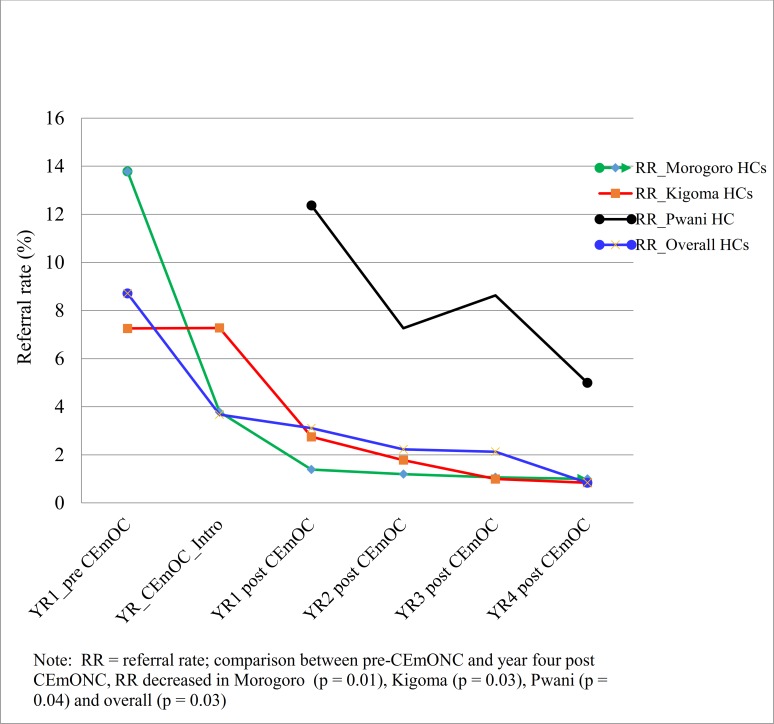
Overtime trends of referral rates before and after introduction of CEmONC services in WLF supported health centres based on regions.

Introduction of CEmONC services in HCs was followed with statistically significant reduction of the proportion of women with obstetric complications who were referred from these facilities to nearby hospitals in all regions (Fig 3). The overall emergency obstetric referral rate decreased by 67% (from 9% to 3%), (F-test = 4.94, p = 0.03).

### Outcome Indicators

**Fig 4:**
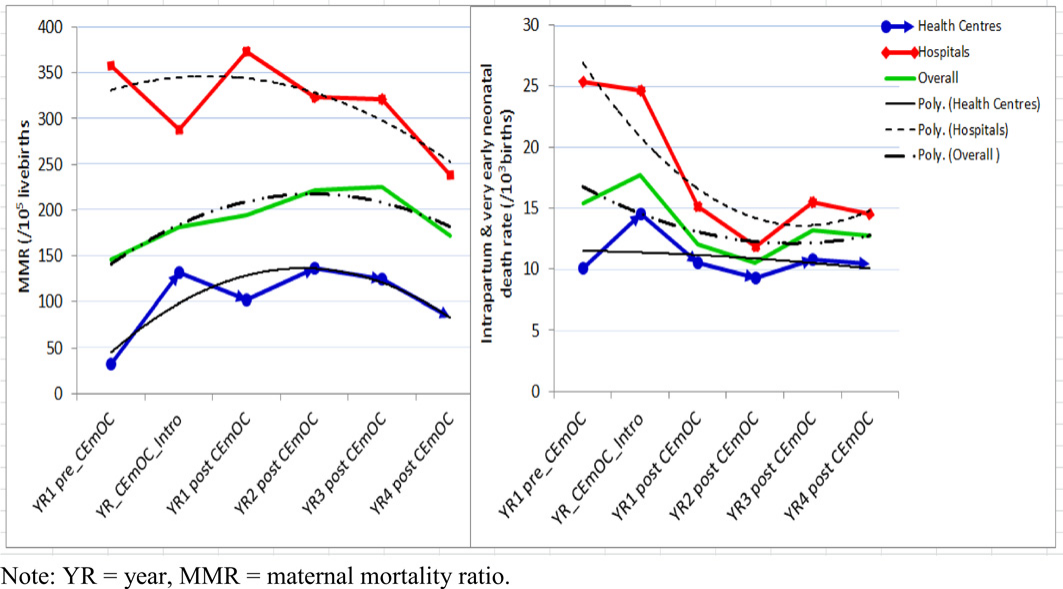
Overtime trend of institutional maternal mortality ratio and intrapartum and very early neonatal mortality rate in WLF supported health facilities before and after introduction of CEmONC services.

During the project period, a total of 190 maternal deaths occurred in all health centres and district hospitals. Of the maternal deaths, only 56 (29%) took place in supported health centres. Overall health centres had statistically significantly lower institutional maternal mortality ratios than district hospitals (F-test = 28.26, p < 0.000). Instead of a decrease, introduction of CEmONC services in health centres was associated with a slight increase of MMR from 32/10^5^ live births before intervention to 83/10^5^ live births in year 4 of the intervention (F-test = 1.82, p = 0.18) (Fig 4). Although there was a trend that MMR decreased overtime in hospitals, there were no statistically significant differences between the pre and post-tests (F-test = 0.90, p = 0.52). It was found that before introduction of CEmONC services, almost all mothers with obstetric complications in these HCs were referred to the district or to other nearby hospitals with CEmONC services. Women with serious obstetric complications, which occurred at community level, often bypassed the HCs and went directly to hospitals. Poor roads and long distances between HCs and CEmONC hospitals as well as financial barriers created delays and ultimately maternal and perinatal deaths and disabilities. Maternal deaths which occurred on the way to the CEmONC facilities were usually not registered in any facilities and therefore were not typically included in maternal mortality figures.

Although, in overall, there were no statistical differences between the pre and post-intervention in health centres, some facilities showed striking results. For example, at Buhingu, a health centre located more than 240 km from the closest CEmONC facility along Lake Tanganyika, five maternal deaths occurred within a period of six months from January to June 2011 before the introduction of CEmONC services. Of these, three were due to ruptured uterus and two due to postpartum haemorrhage leading to a facility based maternal mortality ratio of 2,632/10^5^ live births. After introduction of CEmONC services, from November 2011 to September 2014, only 4 maternal deaths and 920 live births were recorded making a maternal mortality ratio of 435/10^5^ live births.

During the project period, 749 intrapartum and 449 very early neonatal deaths occurred in health centres and hospitals combined. Documentation of IVEND was included in order to shed light on the quality of intrapartum care in the facilities. Of the deaths, 334 (45%) intrapartum and 198 (44%) very early neonatal deaths occurred in the 10 upgraded health centres. Generally, these had statistically significant lower IVEND rates than district hospitals (F-test = 5.89, p = 0.02). Although the trend of IVEND rates tended to decrease, there were no statistically significant differences between the pre and post-interventions in health centres (F-test = 0.77, p = 0.61) as well as in hospitals (F-test = 2.66, p = 0.08) (Fig 4).

From 2011–2014, a total of 131 maternal deaths and 402 IVEND were audited. Of all audited maternal deaths, 89% to 96% were considered avoidable based on the available expertise and resources at the facilities (Table 2). Almost half (44%–45%) of all maternal deaths were attributed to phase 3 delays. More than three quarters (84%–86%) of audited IVEND were considered avoidable. Phase 3 delays contributed to 62%–66% of all avoidable IVEND in these health facilities. IVEND due to severe congenital anomalies, prematurity (birth weight below 1.5 kg) and abruptio placentae were considered unavoidable based on the available expertise and neonatal infrastructure.

**Table 2:** Factors and causes of maternal, intrapartum and very early neonatal deaths in WLF project supported health facilities.

Types of deaths/ causes	Health Centres No. of deaths (% of total)	District hospitals No. deaths (% of total)
**Avoidability of maternal deaths**	38 (100%)	93 (100%)
Unavoidable deaths	4 (11%)	4 (4%)
Delay: phase 1 & 2	13 (34%)	34 (37%)
Delay: phase level 1 & 3	2 (5%)	4 (4%)
Delay: phase 3 (in referring HF)	2 (5%)	10 (10%)
Delay: phase 3	17 (45%)	41 (44%)
**Causes of maternal deaths**	38 (100%)	93 (100%)
Obstetric haemorrhage	14 (37%)	21 (23%)
Pre- eclampsia & Eclampsia	6 (16%)	11 (12%)
Complications of obstructed labour	7 (18%)	19 (20%)
Puerperal sepsis	2 (5%)	15 (16%)
Severe anaemia	3 (8%)	9 (10%)
Sudden death syndrome*	5 (13%)	5 (5%)
HIV & AIDS in pregnancy	0 (0%)	4 (4%)
Others causes†	1 (3%)	9 (10%)
**Avoidability of IVEND (total audited)**	204 (100%)	198 (100%)
Unavoidable deaths	28 (14%)	31 (16%)
Delay: phase 1 & 2	28 (14%)	29 (15%)
Delay: phase 1 & 3	7 (3%)	4 (2%)
Delay: phase 3 (in referring HF)	6 (3%)	11 (6%)
Delay: phase 3	135 (66%)	123 (62%)
**Causes of IVEND**	204 (100%)	198(100%)
Prolonged/obstructive reasons††	133 (65%)	141 (71%)
Complications of breech delivery	22 (11%)	11 (6%)
Cord prolapse	8 (4%)	10 (6%)
Prematurity	19 (9%)	18 (9%)
Congenital anomalies	7 (3%)	4 (2%)
Antepartum haemorrhage	7 (3%)	6 (3%)
Other causes	8 (4%)	8 (4%)

Note

* Sudden death syndrome included death on the operating table due to various complications like anaesthesia and embolism

† Other causes of maternal deaths included malaria in pregnancy, postpartum cardiomyopathy, sickle cell crisis, complications of abortion;

†† Prolonged/obstructive reasons included birth asphyxia due to obstructed labour, cord around the neck and retained second twin, compound presentation and other related reasons; (2) delays at phase 1 & 2 were combined because it was not possible to exactly know whether the mother had delayed in decision making or reaching the facility. Other causes of IVEND included complications of pre and eclampsia, infection, hypoglycaemia, etc.

During the project period (2009–2014) a total of 387 women with eclampsia, 709 with postpartum haemorrhage and 261 with ruptured uterus were reported in supported health centres and hospitals. Specific case fatality rates for eclampsia, severe postpartum haemorrhage and ruptured uterus ranged between 3.3% and 6.7% in health centres, and ranged between 5.4% and 9.7% in district hospitals (Table 3). From 2011, when audit was introduced, a total of 87 and 105 women with ruptured uterus were registered in health centres and hospitals respectively. Of these women with ruptured uterus, 51 cases (59%) in the health centres and 65 cases (62%) in district hospitals had occurred before admission. Because of inadequate documentation, the place where uterine rupture had occurred was not established in 20 cases (23%) in the health centres and 23 cases (22%) in hospitals.

**Table 3:** Case fatality rates in WLF project supported CEmONC health facilities.

	Number of Cases	Number of Maternal Deaths	Specific Case Fatality Rate
**Eclampsia**			
Health Centres	159	6	3.8
District Hospitals	228	13	5.7
**Postpartum Haemorrhage**			
Health Centres	360	12	3.3
District Hospitals	349	19	5.4
**Ruptured Uterus**			
Health Centres	105	7	6.7
District Hospitals	156	15	9.6

Note: There was under documentation of cases as some women with morbidities who did not present with features of organ failure were not registered in the delivery records books.

## Discussion

Proving which interventions work to address maternal and perinatal mortality in the underserved rural areas in resource limited settings has remained a challenge for decades [7,23]. This article provides a body of evidence on a set of interventions implemented in rural areas that has shown promising results. This study showed that upgrading and equipping health centres and training and supporting associate clinicians to provide CEmONC is associated with statistically significant increases in the number of women coming to deliver in facilities, reductions in obstetric referral rates and remarkably increased population-based CS rates.

### CEmONC Services Task-Sharing

Due to the acute shortage of doctors, most African countries, Tanzania inclusive, have a set of associate clinicians who are neither university graduate doctors nor nurses but provide clinical care [24]. Unlike other initiatives, in this project, associate clinicians were trained not only in CEmONC but also in anaesthesia. Like many other reports, these findings suggest that task-sharing by training, deploying, and supporting associate clinicians to provide CEmONC services is one of the key interventions that could have a positive impact on maternal and perinatal health in remote rural areas [24,25,26,27]. Our findings strongly suggest that CEmONC task-sharing as a means of alleviating the provider gap blended with necessary infrastructure development in healthcare facilities in remote and underserved areas of sub-Saharan Africa can lead to consistent availability of adequate CEmONC at the local level and an increase in EmONC met need. Such reorganization of associate clinicians makes more efficient use of the human resources already available and quickly increases capacity to decentralize CEmONC, and sounds to be a viable solution for improving maternal health care coverage [28].

### Impact of the Interventions: CEmONC Services Utilization

Our findings indicate that once the availability of CEmONC services becomes known in the population, the number of women going to the health facility increases and thus reduces first phase delays. The proportion of all births in EmONC facilities reached 128% because many expectant mothers from other ‘wards’ (outside the catchment area) also began using supported facilities. The impressive utilization of these services found in this project lends support to the idea that if quality emergency obstetric services are made available, many women will use them [29,30]. The variability of the change in utilization of services could have been due to various local factors such as catchment population size, the availability of CEmONC services outside the catchment areas, as well as other sociodemographic variables.

Although this data should be interpreted with caution, it can be reasonably assumed that the proportion of EmOC births in the catchment populations included also the majority of women with obstetric complications who especially needed CEmONC services. This is based on the assumption that only around 15% of pregnancies in any population usually develop obstetric complications [10,31]. Delivery in EmONC facility is a key element for ensuring timely intervention when needed and for reducing the risk of maternal and perinatal death among women delivering with obstetric complications. Although, increased numbers of women giving births in CEmONC health facilities may not necessarily equate high-quality care, such a remarkable increase suggests that women with obstetric complications believed the services to be of reasonable quality.

Introduction of CEmONC services in HCs led to a statistically significant reduction of obstetric referrals from these facilities to hospitals, from 9% to 3%, (p = 0.03). The fact that referral rates of women with obstetric complications decreased significantly and that the rural health centres’ catchment population-based CS rates reached 9%, strongly suggest that a significant proportion of women who needed CS received these services. Unmet need for CS thus decreased significantly. It has been reported elsewhere that in places where maternal mortality is high, the rate of CS tends to be lower [10]. These results strongly suggest that accessibility to life saving caesarean section services can be improved even in marginalized and underserved rural communities by upgrading health centres and deploying associate clinicians. However, the importance of close monitoring, supervision and mentoring by expert clinicians to ensure good-quality care must not be underestimated [32].

### Impact of the Interventions: Obstetric Outcomes

Introduction of CEmONC services in health centres was not associated with any significant change of MMR before and after the intervention. As expected the trend of MMR tended to decrease overtime in hospitals, in overall, although there were no statistically significant differences between the pre and post-tests. Although not statistically significant, the overall IVEND rate decreased from 17/10^3^ live births to as low as 11/10^3^ live births two years after introduction of CEmONC services in these health centres (p = 0.61). The failure to statistically decrease MMR and IVEND in health centres can partly be explained by a number of facts noted before interventions; 1) under reporting of maternal and IVEND deaths; 2) a practice of evacuating all women with obstetric complications from health centres leading to very low facility-based maternal and perinatal mortality rates; 3) low utilization of delivery services in these facilities by women who developed obstetric complications at home before the interventions. Prior to CEmONC services a large number of women with obstetric complications would bypass health centres as they knew that the facilities were severely limited in the types of lifesaving services available. As a result, there were frequent reports of maternal and perinatal deaths which were attributed to lack of accessibility of CEmONC services. Because of poor roads, long distances, financial and/ or transport problems some of those mothers who bypassed and those who were referred from these health centres to the CEmONC facilities died or lost their babies before or even after reaching these facilities. Some of these women with obstetric complications arrived at the CEmONC facilities in a moribund state too late for successful intervention.

Taking into account those women with obstetric complications who bypassed these health centres and those who were referred but died on the way or after reaching the referral facilities during the pre-CEmONC period and did not appear in any statistics, these findings suggest that there could have been significant reduction of MMR and IVEND. These findings suggest that the impact on obstetric outcomes after introducing CEmONC services in health centres could better be assessed using community-based rather than facility-based maternal and perinatal mortality figures. In the district hospitals IVEND-rates decreased by more than 50% from 25/10^3^ live births to as low as 12/10^3^ live births (p = 0.08). All these findings suggest that upgrading of health centres to enable them to provide comprehensive EmOC is a promising intervention.

The impact of this project can strongly be attributed to enhancing facility-based clinical care using trained associate clinicians to provide CEmONC services. It has also been reported that strengthening skilled birth attendance is one of the few high-impact health sector interventions which explain why some countries have achieved faster reductions of maternal and perinatal mortality compared to others [33]. Considering that the majority of maternal deaths are caused by direct obstetric conditions which occur around the time of giving birth, and the fact that 84%–96% of all maternal deaths and IVEND were considered avoidable, there is a need for strengthened supportive supervision, clinical audit and on the job training in clinical decision making and clinical techniques [31,34,35] Delivery with equipped skilled attendance enhances prompt and timely intervention in the case of life-threatening complications.

### Impact of the Interventions: CEmONC Referral Costs

Reduction of obstetric referrals implied reduced phase two delays and referral costs for the facilities and decreased clients’ costs for accessing CEmONC services. One specific example comes from Buhingu where there was no ambulance for referral services. Prior to the introduction of CEmONC services at Buhingu, a mother with an obstetric complication was required to wait up to 18 hours for the public boat to transport her along Lake Tanganyika to Maweni hospital in Kigoma town; the alternative would have been to pay approximately $1,250 USD to hire a private boat. Another example comes from Mlimba where the HC is located 150 km from the closest CEmONC facility. Between January and June 2010, the district council spent $ 13,010 USD transporting 102 mothers with obstetric complications from Mlimba HC to the closest CEmONC hospital. Like many other places in sub-Saharan Africa, inadequate inter-facility transportation for referral, high costs of private transportation, poverty and lack of women autonomy seriously affect the outcomes of women with obstetric complications [36,37]. Our findings suggest that decentralization of CEmONC services reduces individual and facility-based transport costs and may also contribute to improved pregnancy outcome by reduced phase two delays.

These results indicate that upgrading extremely remote rural health centres to provide CEmONC services is an appropriate and effective intervention and it has proven feasible and acceptable. Like other programs our findings indicate that establishing modest surgical theatres and training associate clinicians in life-saving skills, can enable lower-level facilities to cope with serious obstetric emergencies [29,30]. This report is an inspiring testament to governments and all international development partners on how to advance maternal and perinatal care in the most isolated remote and rural areas in resource limited settings.

### Factors for Change

Like many other programs, the provision of CEmONC services and quality of care 24/7 in upgraded health centres was hugely affected by several factors: 1) lack of regular replacements of essential supplies and drugs from the government; 2) shortage of staff; 3) lack of incentives for health providers including on call allowances for work outside the regular work hours; and 4) inadequate management of human resources for health leading to unnecessary absenteeism [38]. These factors contributed to unnecessary referrals even for cases which could have been managed in upgraded health centres. In an attempt to address these factors, the project team regularly shared project results and challenges with policy makers, administrative authorities and politicians from district, regional and national levels. Like other reports these findings suggest a need for changing relevant policies on distribution of essential supplies, drugs, deployment of trained workforce and continuous sensitization of key local government officials and influential community leaders in order to sustain services and reduce phase three delays [30].

### Limitation of study

Women seeking services often do not see catchment area boundaries and may seek facility-based maternity services outside their areas. This can affect the precision of population based indicators. Therefore, population-based CS rates in this article should be interpreted with caution. In addition, the existence of other possible factors not identified by the authors may have contributed to the change in utilization of services in health centres. It is also likely that the number of maternal deaths and cases of morbidity in supported facilities were underestimated due to problems with record keeping at facilities. There was also a methodological limitation as there were no controls for comparison purposes.

## Conclusions

Upgrading health centres to provide CEmONC services in underserved rural areas and using locally available associate clinicians has been shown to increase facility delivery rates, reduce phase two delays and referral costs, improve population based C-section rate and pregnancy outcomes. Further, by using existing resources, project interventions are more likely to be sustainable and replicable. The authors, therefore, recommend that Ministries of Health in other resource limited countries strongly consider using the findings and experiences presented in this article to design and implement similar projects to improve maternal and perinatal health in their countries.

## Supporting Information

S1 Appendix WLF supported health facilities’ performance dataset. (XLSX)
